# Severity of Peri-ictal Respiratory Dysfunction With Epilepsy Duration and Patient Age at Epilepsy Onset

**DOI:** 10.3389/fneur.2020.618841

**Published:** 2020-12-18

**Authors:** Kiran Kanth, Katherine Park, Masud Seyal

**Affiliations:** Department of Neurology, University of California, Davis, Sacramento, CA, United States

**Keywords:** SUDEP, seizure, epilepsy duration, respiration, apnea

## Abstract

Respiratory dysfunction preceding death is fundamental in sudden unexpected death in epilepsy (SUDEP) pathophysiology. Hypoxia occurs with one-third of seizures. In temporal lobe epilepsy, there is volume loss in brainstem regions involved in autonomic control and increasing neuropathological changes with duration of epilepsy suggesting increasingly impaired regulation of ventilation. In animal models, recurrent hypoxic episodes induce long-term facilitation (LTF) of ventilatory function, however, LTF is less robust in older animals. LTF of ventilation may, to some degree, ameliorate the deleterious effects of progressive brainstem atrophy. We investigated the possibility that the duration of epilepsy, or age at epilepsy onset, may impact the severity of seizure-associated respiratory dysfunction. Patients with focal epilepsy undergoing video-EEG telemetry in the epilepsy monitoring unit (EMU) were studied. We found a significant relationship between age at epilepsy onset and duration of peri-ictal oxygen desaturation for focal seizures not progressing to bilateral tonic-clonic seizures, with longer duration of peri-ictal oxygen desaturation in patients with epilepsy onset at an older age but no significant relationships between duration of epilepsy or age at EMU admission and ventilatory dysfunction. Our findings suggest an intriguing possibility that LTF of ventilation may be protective when epilepsy starts at a younger age.

## Introduction

Sudden unexpected death in epilepsy (SUDEP) is a common cause of epilepsy related mortality (1–5). Respiratory dysfunction preceding death is implicated as fundamental in SUDEP pathophysiology (6). Ictal apnea, hypoxemia and hypercapnia frequently occur in the ictal and postictal period (7, 8). It has not been established whether the duration of epilepsy or age at epilepsy onset might influence the severity of seizure-related apnea or hypoxia. Imaging studies, in patients who subsequently die of SUDEP, show alterations in brain regions essential for cardiorespiratory recovery and respiratory patterning (9) that may negatively influence the severity of peri-ictal respiratory dysfunction. Physiological studies in animals indicate that recurrent hypoxic episodes result in long-term facilitation (LTF) of respiratory function (10, 11). The degree of LTF with recurrent hypoxia is reduced in older animals (10, 11). In patients with epilepsy and recurrent ictal hypoxic episodes, LTF may provide protection by reducing the severity of respiratory dysfunction with uncontrolled seizures.

We investigated ictal-related respiratory dysfunction in patients admitted to the epilepsy monitoring unit (EMU). The aim of this study was to investigate whether the duration of epilepsy, age at epilepsy onset or age on admission to the EMU were associated with the severity of ictal-associated respiratory dysfunction.

## Materials and Methods

Patients with pharmacoresistant focal epilepsy undergoing scalp electroencephalography (EEG) monitoring in the EMU were studied. Details of video-EEG and respiratory function monitoring in the EMU have been described previously (7). Focal seizures without progression to bilateral tonic-clonic seizures as well as focal seizures that progressed to bilateral tonic-clonic seizures (FBTCS) were analyzed. We considered that there may be a floor/ceiling effect on the depth and duration of peri-ictal oxygen desaturation with FBTCS. Such effects might mask any age-related changes, and therefore, we performed an analysis of focal seizures that did not progress to bilateral tonic-clonic seizures.

Patients in this EMU have synchronized recordings of nasal airflow, abdominal excursions, and digital pulse oximetry during video-EEG telemetry monitoring. Patients were selected for analysis if a usable nasal airflow signal, respiration-related abdominal excursion signal, or digital pulse oximetry, was available during recorded seizures. Apnea was defined as cessation of either the nasal airflow or respiratory abdominal excursion signal for 5 s or longer during a recorded seizure. The end of apnea was defined by the onset of regular respirations and cessation of apneic events of 5 s or longer. Oxygen desaturation nadir and duration of oxygen desaturation <90%, as determined by pulse oximetry, were evaluated. In patients where more than one seizure was recorded, the mean apnea duration, mean oxygen desaturation nadir, and the mean desaturation duration was determined for that individual and those values used for subsequent analysis. Age at epilepsy onset, duration of epilepsy, and age at EMU admission were recorded in years. Seizure duration for all seizures and seizures without progression to bilateral tonic-clonic seizures was recorded. The retrospective study was approved by the University of California, Davis Institutional Review Board. Statistical analysis was performed using Sigmaplot. Linear regression analysis was used to determine whether there was a relationship between age at epilepsy onset, duration of epilepsy, or age at EMU admission and the various measures of peri-ictal respiratory dysfunction. Statistical significance was defined as a *p* < 0.05. For significant associations determined on simple linear regressions analysis, a Pearson Product Moment Correlation was performed. To account for multiple comparisons, a multivariable linear regression analysis was performed for possible predictors of respiratory dysfunction that included associations with significance levels of *p* ≤ 0.2 on simple linear regression. A Fisher Exact Test was performed to compare the proportion of temporal and extratemporal seizures in childhood (<21 years) vs. adult onset epilepsy.

## Results

Data from 73 patients (37 female) were analyzed. Relevant clinical information is shown in Table 1. Data on patient age, age at epilepsy onset, and epilepsy duration and relevant analyses of peri-ictal respiratory dysfunction are shown in Table 2.

**Table 1:** Clinical characteristics.

**Patient gender**	37 female 36 male
**Seizure frequency at EMU evaluation (seizures/month)**	Mean 17.2 Median 7.5 Range 0.33–180
**Number of antiseizure medications**	Mean 1.9 Median 2 Range 0–3
**Focal seizure classification**	**Number of patients**
Focal aware seizures	43
Focal unaware seizures	177
**Lobar location of epilepsy**	**Number of patients**
Temporal	54
Frontal	8
Parietal	1
Occipital	2
Multifocal	2
Undetermined	6
**Etiology of epilepsy**	**Number of patients**
Mesial Temporal Sclerosis	21
Focal Cortical Dysplasia	3
Neuronal Migration Disorder	2
Neoplasm	7
Traumatic Brain Injury	5
Vascular Malformation	2
Infection	1
Other Structural	4
Unknown	28
**Seizure Onset lateralization**	**Number of Seizures**
Bilateral	21
Left	196
Right	204
Unknown	75

**Table 2:** Summary data including all focal and FBTC seizures.

	**Mean**	**Median**	**Range**
Age at epilepsy onset (Years)	14.2	12	0–61
Age at EMU admission (Years)	35.9	34	16–66
Duration of epilepsy at EMU admission (Years)	22.3	18	1–52
Mean desaturation duration (Seconds)	74.6	62	7–347
Mean oxygen desaturation nadir (%)	84.8	87	58–99
Mean apnea duration (Seconds)	50.4	40	10–180
Mean seizure duration (focal seizures only) (Seconds)	89.6	72	7.4–360
Mean seizure duration (all seizures) (Seconds)	97.7	80	8.1–376

**Figure 1:**
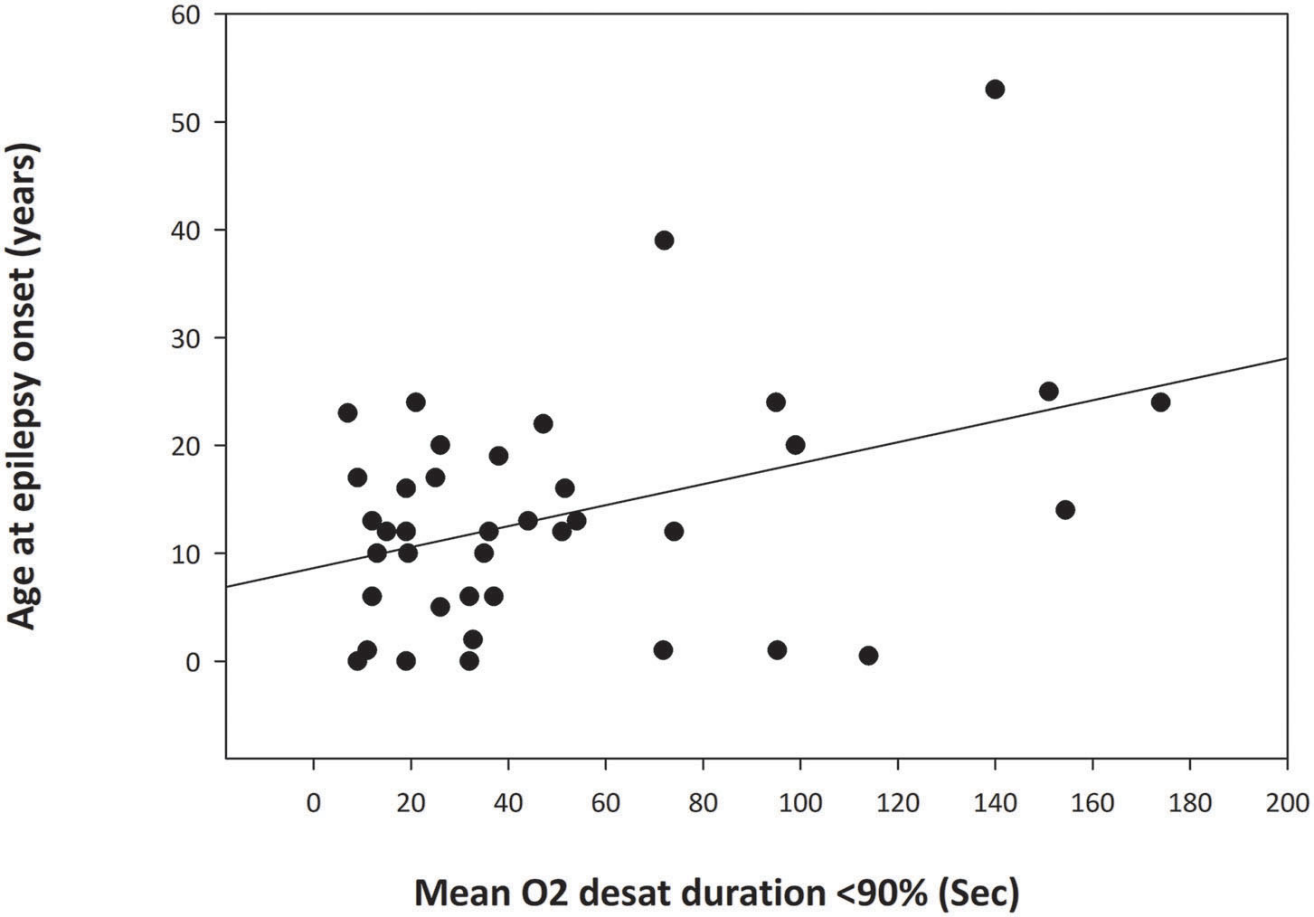
Simple Linear Regression analysis graph demonstrating relationship between the age at epilepsy onset and duration of oxygen desaturation of <90% in focal seizures without secondary generalization (*p* = 0.012). The abscissa indicates the mean duration of postictal oxygen desaturation below 90% in seconds. The ordinate is the patient age at onset of epilepsy in years. The filled circles are data points for each patient. The solid line through the data points is the regression line.

There was a significant relationship (*p* = 0.012) between age at epilepsy onset and duration of peri-ictal oxygen desaturation for focal seizures that did not progress to bilateral tonic-clonic seizures (BTCS) (Figure 1) with greater duration of peri-ictal oxygen desaturation in patients with older age at epilepsy onset (Simple regression analysis: Mean O_2_ desaturation duration (seconds) = 28.91 + (1.632 ^*^ Age (years) at epilepsy onset). Pearson correlation coefficient 0.399, *p* = 0.012. On multiple linear regression analysis for age at epilepsy onset, duration of epilepsy and age at EMU admission, only the age at epilepsy onset was significantly associated with oxygen desaturation duration (*p* = 0.045).

Seizure duration, for all seizures or focal only seizures, was not associated (*p* > 0.1) with duration of epilepsy, age on EMU admission or age at epilepsy onset. There were no significant relationships (*p* = > 0.1) between duration of epilepsy or age at EMU admission and the various measures of peri-ictal dysfunction (oxygen desaturation nadir, duration of desaturation below 90% and apnea duration for focal seizures with or without progression to BTCS. Oxygen desaturation nadir was significantly associated with the duration of oxygen desaturation (*p* < 0.001). Reported seizure frequency at EMU admission was not associated with the duration of postictal oxygen desaturation. There was no significant difference (*p* = 0.095) in the proportion of temporal vs. extratemporal seizures comparing childhood onset epilepsy (<21 years) vs. patients where seizures started in adulthood (≥21 years).

Oxygen desaturation nadir data were available for 71 patients, apnea duration data were available for 30 patients, and data for duration of oxygen desaturation below 90% were available for 57 patients.

## Discussion

We have shown that there is a significant relationship between the age at epilepsy onset and the duration of peri-ictal oxygen desaturation. An older age at epilepsy onset is related to a more prolonged period of seizure-related oxygen desaturation. Our data suggest that the age of the patient at epilepsy onset is unrelated to the initial severity of peri-ictal oxygen desaturation as indicated by the oxygen desaturation nadir. There is, however, significantly delayed recovery of respiratory function in patients with older age at epilepsy onset. The duration of epilepsy was not related to the severity of respiratory dysfunction.

Our findings cannot be accounted for by the possibility that seizure durations are longer when seizures start at an older age. We had previously shown that the duration of individual seizures was directly associated with the degree and duration of peri-ictal oxygen desaturation (7, 8). In the present study we find that seizure duration is not related to the age at epilepsy onset or duration of epilepsy. These findings make it unlikely that the longer duration of postictal respiratory dysfunction, at an older age of epilepsy, onset is a consequence of longer seizure duration.

MRI studies in patients with temporal lobe epilepsy demonstrate volume loss in the brainstem regions considered important in autonomic control (12, 13). These alterations are more pronounced in patients dying of SUDEP (12, 13). It has been hypothesized that recurrent seizure-related hypoxic events may cause increasing damage to these brainstem regions (12, 13). Immunohistochemical studies of the ventrolateral medulla demonstrate reductions in neuromodulatory neuropeptides and monoaminergic systems in patients dying of SUDEP relative to control (14), and there was an association of neuropathological alterations with duration of seizures (14). As such, one may expect to find increasing seizure-related respiratory dysfunction with longer duration of uncontrolled epilepsy, however, this was not observed in the present study.

Hypoxic episodes with oxygen desaturation to <90% occur in about one-third of focal onset seizures (7). There is convincing evidence for persistent long-term facilitation (LTF) of respiratory activity following recurrent hypoxic episodes (10, 15–19). It is conceivable that LTF of respiratory function with uncontrolled seizures and hypoxic events may, to some degree, counteract the potentially deleterious effects of seizure-related brainstem atrophy and reductions in neuromodulatory neuropeptides considered important in respiratory control. These brainstem LTF changes may account for the fact that no significant peri-ictal respiratory disturbance with duration of epilepsy was detected in our group of patients. In animal models, exposure to intermittent hypoxia results in augmentation of respiratory activity that can persist for, at least, many hours after hypoxic episodes are ended (10). LTF has been demonstrated for phrenic nerve activity in rodents under anesthesia and LTF of ventilation occurs in awake animals (15, 16). LTF can be induced by hypoxic episode durations similar to those that occur in obstructive sleep apnea (17, 18). LTF appears dependent on changes in mitochondrial ultrastructure and increased cytochrome oxidase activity in the pre-Bötzinger nucleus, a structure in the ventrolateral medulla essential for the generation of respiratory rhythms (19). The mitochondrial alterations suggest that the effects of hypoxia are robust and persistent.

In the absence of recurrent hypoxic events, however, any post-ictal LTF of respiratory function would be expected to diminish with time following a seizure-related hypoxic event with an unknown decay rate. LTF, if present, may have a greater effect on respiratory function in patients with frequent seizures than in those with rare events.

Limitations of this retrospective study include the lack of a reliable estimate of seizure frequency since epilepsy onset and, perhaps more importantly, the frequency of seizure-related hypoxic events prior to EMU admission. These are potentially important factors that could, by cumulative damage to respiratory networks, progressively influence the severity of respiratory dysfunction over extended periods of time. Investigation of these issues in future prospective studies is warranted.

Of particular interest to the findings of our study is the observation that, in animal models, old age results in loss of LTF, whereas younger age is associated with greater LTF (11). Recent population-based cohort studies indicate that SUDEP incidence is similar in all age groups including patients <16 years old and those older than 50 years (4). The weighting of factors contributing to BTCS and SUDEP may vary across the age spectrum. More profound respiratory dysfunction in the peri-ictal period contributing to SUDEP may be of greater concern when epilepsy starts at an older age. However, while intriguing, the relevance of our findings with regards to SUDEP pathophysiology remains speculative.

## Data Availability Statement

The raw data supporting the conclusions of this article will be made available by the authors, without undue reservation.

## Ethics Statement

The studies involving human participants were reviewed and approved by University of California, Davis. Written informed consent for participation was not required for this study in accordance with the national legislation and the institutional requirements.

## Author Contributions

KK jointly conceived the study, contributed to design of study, writing of manuscript, and manuscript revisions. KP contributed to writing of manuscript and manuscript revisions. MS conceived the study, wrote the initial draft, performed data analysis, and contributed to manuscript revisions. All authors contributed to the article and approved the submitted version.

## Conflict of Interest

The authors declare that the research was conducted in the absence of any commercial or financial relationships that could be construed as a potential conflict of interest.
